# Personal

**Published:** 1888-08

**Authors:** 


### Personal.

Mr. Lawson Tait, F. R. C. S. E., Professor of Gynecology, Queen's College, Birmingham, England, who visited Albany a few years since and is so kindly remembered by many here, had at the last commencement of Union College the honorary degree of LL.D. conferred upon him by the Board of Trustees. All will agree that this is an honor worthily bestowed.—*Albany Medical Annals, July.*

General Sheridan's case has, it is universally admitted, been brilliantly managed by his attending physicians. There is some satisfaction in finding that a case which has been so conspicuously before the public is one that illustrates the resources of our art. Certainly, if it were not for medical skill, including careful nursing with the use of powerful drugs, General Sheridan would not have lived so long as he has done.—*Medical Record.*

